# Immunofluorescent Localisation of Human Alpha Fetoprotein in Fetal and Neonatal Livers and Cultured Cells from Hepatocellular Carcinoma

**DOI:** 10.1038/bjc.1971.44

**Published:** 1971-06

**Authors:** J. A. Smith, T. I. Francis, G. M. Edington, A. O. Williams

## Abstract

**Images:**


### IMMUNOFLUORESCENT LOCALISATION OF HUMAN ALPHA FETOPROTEIN IN FETAL AND NEONATAL LIVERS AND CULTURED CELLS FROM HEPATOCELLULAR CARCINOMA

J. A. SMITH*, T. I. FRANCIS†, G. M. EDINGTON* AND A. O. WILLIAMS*

*From the Departments of Pathology* (Morbid Anatomy) and of Medicine,†*
*University of Ibadan, University College Hospital, Ibadan*

Received for publication, January 1, 1971

**SUMMARY.—The indirect immunofluorescence technique demonstrated that human alpha fetoprotein (AFP) was present in a focal pattern in the cytoplasm of malignant liver cells of patients whose sera contained AFP. A few fibroblast-like cells in tissue culture of liver biopsies from patients with hepato-cellular carcinoma and AFP in their sera also had the protein. The intracellular localisation of human AFP was confirmed by centrifugation of washed, homogenised and ultra-sonically disrupted neonatal liver cells. Examination of livers of fetuses and neonates showed that AFP was present predominantly in the periportal parenchymal cells.**

ALPHA fetoprotein (AFP) is known to be secreted by the fetal liver of mice, rats and man (Abelev and Bakirov, 1967; Luria *et al.*, 1969; Van Furth and Adinolfi, 1969). It is produced by transplantable mouse hepatoma (Irlin *et al.*, 1966). Tatarinov (1965*a*) demonstrated the appearance of AFP in the serum of some patients with primary liver cell carcinoma and teratocarcinoma. Using the agar double diffusion technique, less than 50% of hepatocellular carcinomas in Europe and between 70 and 80% in some parts of Africa were shown to produce serum AFP (Foli *et al.*, 1969; Sankale *et al.*, 1970). Why some hepatomas secrete AFP and others do not is unknown. In this study, an attempt has been made to localise the site of production of AFP by immunofluorescence and at the subcellular level by centrifugation. The sensitivities of the immunofluorescent and the Ouchterlony techniques have also been compared.

### MATERIALS AND METHODS

*Liver.*—Portions of liver from a 28 weeks aborted fetus, a 5 hours autopsied neonate, eight necropsied patients five of whom had AFP in their sera, were fixed in 10% formalin for conventional histology. Other portions from each source were snap-frozen in liquid nitrogen and stored at −70° C. These tissues were obtained within 48 hours of death and stored for up to 1 week in some cases before use.

*Antiserum.*—Rabbit antiserum to human AFP was obtained by the method of Masopust *et al.* (1968). It was made monospecific according to the method of Abelev *et al.* (1967). The total globulin from 10 ml. of the monospecific rabbit antiserum was precipitated with 10 ml. of saturated $(NH_4)_2SO_4$. The precipitate

Request for reprints should be directed to Dr. J. A. Smith.

was dissolved in 4 ml. phosphate buffered saline (PBS) at pH 7·5 and dialysed against the PBS for 24 hours at 4° C. The solution was afterwards concentrated to 2 ml. by ultrafiltration at 4° C. It was absorbed for 20 minutes at 4° C. with human liver powder prepared according to the method of Holborow and Johnson (1967).

*Immunofluorescence.*—Cryostat sections were cut at 4 $\mu$. They were dried with a hair drier. Two drops of absorbed rabbit antiserum were layered on the section and allowed to react at room temperature (RT) for 30 minutes in a moist chamber. The antiserum was washed off with PBS for 30 minutes under magnetic stirring. It was changed once. Two drops of fluorescein conjugated swine anti-rabbit globulin (Conjugate made by Institute of Sera and Vaccines, Praha, Czechoslovakia) previously absorbed with guinea-pig and human liver powders were then put on the sections for 30 minutes. The conjugate was also put on control slides to which antiserum had not been added. The conjugate had previously been tested by double diffusion for monospecificity to the rabbit antiserum. It gave only one precipitin line. The slides were again washed with PBS for 30 minutes with one change half way through. Ten per cent glycerine was used as mountant. The preparations were examined with a fluorescent microscope using HBO 200 mercury vapour lamp as source, BG 12 primary and 490 m$\mu$ secondary filters. Serial cryostat sections of the liver stained for immunofluorescent studies were also stained with H. and E. and Giemsa respectively. Comparison of sections from each liver studied was made to correlate areas of positive specific fluorescence with the same areas as seen on conventional histology. Also after the immunofluorescent examination, the same tissue culture slides and cryostat sections were again stained with Giemsa.

*Tissue culture.*—Human liver cells were cultured by a modification of the methods of Zuckerman *et al.* (1967); Zuckerman (1967); Sullman *et al.* (1968) and Taylor *et al.* (1969). Tissues were obtained either from open surgical or closed needle biopsies. They were cut into several minute pieces and initially trypsinised with 0·25% trypsin for 30 minutes before culturing in ring chambers on chicken plasma clot. It was later found unnecessary to trypsinise or to use plasma clot. Culture medium consisted of TC 199, 20% human serum, 1% chicken embryo extract, neomycin 33 units/ml., and mycostatin 125 units/ml. Incubation was at 37° C. Carbon dioxide atmosphere was not used. After various intervals ranging from 18 to 141 days fibroblast-like cells, which could not be subcultured (Zuckerman, 1970, personal communication), were harvested. The harvested slides and coverslips were washed with PBS for 2 hours and dried overnight at RT (25° C.). The same procedure of indirect immunofluorescent staining as described above was applied. The complete medium and its individual components were tested against rabbit antiserum by double diffusion in agar. No precipitin lines were produced even after ten fold concentration.

The origin of the liver biopsies cultured were from patients with diseases shown in Table I. At the time of examination the cells had been in culture for varying periods as shown also in Table I.

*Homogenisation.*—Fragments of 20 g. fetal liver were cut into small pieces and washed with PBS until free of visible blood. The pieces of tissue were homogenised with 5 ml. PBS in a hand glass homogeniser. Part of the homogenate was centrifuged at 750 *g* and RT for 5 minutes. Three zones were obtained. The upper two were greyish and the deposit red. The first two zones were pooled

TABLE I.—*Summary of Data on Tissue Culture Cells from 13 Liver Biopsies*

| Diagnosis | Number of patients | Sex M | Sex F | Age in years | Duration of culture in days | IMF test for AFP |
|---|---|---|---|---|---|---|
| (1) AFP positive hepatoma | 4 | 3 | 1 | 24–50 | 19, 26, 50, 50 | ++ ++ |
| (2) AFP negative hepatoma | 1 | 1 | – | 45 | 141 | — |
| (3) Hepatic metastasis of gastric carcinoma | 2 | 1 | 1 | 45, 65 | 18, 82 | — |
| (4) Chronic duodenal ulcer | 2 | 2 | – | 24, 30 | 109 | — — |
| (5) Post necrotic cirrhosis of the liver | 1 | 1 | – | 35 | 26 | — |
| (6) Chronic active hepatitis | 1 | 1 | – | 40 | 66 | — |
| (7) Kwashiorkor | 1 | .. | 1 | 6 | 41 | — |
| (8) Secondary biliary cirrhosis | 1 | 1 | – | 3/12 | 67 | — |

together and labelled supernatant I. The two samples were examined with the phase contrast microscope. Supernatant I was centrifuged at 800 *g* and 4° C. for 30 minutes. Two zones were again obtained, a supernatant and a deposit. An equal volume of PBS was added to the deposit and centrifuged at 800 *g* and 4° C. for 30 minutes. This procedure was continued until the supernatant, after ten-fold concentration by ultrafiltration, no longer reacted with rabbit antiserum to AFP by double diffusion. The deposit of cells was then disrupted by ultrasonication and centrifuged according to the method of Williams and McFarlane (1968). The zones obtained were concentrated a ten fold by ultrafiltration and tested for AFP by double diffusion.

### RESULTS

### *Immunofluorescence*

(a) *Cryostat sections.*—In the fetal and neonatal liver most of the immunofluorescence was periportal in localisation. All the hepatocytes of the limiting plate, one or two cells thick, around the portal tract showed fluorescence. Only occasional hepatocytes showed fluorescence in other parts of the liver lobule. The fluorescence in each positive cell was homogeneous and limited to the cytoplasm (Fig. 1). In the liver of AFP seropositive hepatocellular carcinoma, fluorescence was patchy in distribution. It was present either in single cells or in a clump of cells usually not more than five in a neoplastic area. In a low power field (×70 magnification) the total number of fluorescent cells was never more than 20% of all the cells seen in the field. The fluorescence was also homogeneous and cytoplasmic as in fetal cells. There was no fluorescence in any hepatocyte from AFP negative hepatocellular carcinomas or the livers of those that died of other diseases (Fig. 2). Furthermore there was no fluorescence in the non-malignant hepatocytes of the AFP positive primary liver cell carcinomas. Sometimes fluorescence along the sinusoids focally in both the controls and hepatoma patients was seen (Fig. 2). Some cytoplasmic granular orange autofluorescent spots were sometimes seen in the cytoplasm of the non-malignant hepatocytes (Fig. 2).

(b) *Tissue culture.*—There was marked fluorescence of the extracellular granular debris which presumably was not removed by washing. There was cytoplasmic staining of a few fibroblast-like cells only in AFP positive hepatocellular carcinoma cultures but the numbers were very few, only one or sometimes none being seen per low power field (Fig. 3). At least one positive cell was seen in each of the four AFP positive liver cell carcinoma cultures. No cytoplasmic fluorescence was seen in any of the other cultures although some staining of the cell margins was noted especially in two cultures. The only AFP negative hepatoma, which had also been in culture for the longest period did not show fluorescence. It was not possible to say whether the number of cells showing fluorescence decreased, increased or remained static with time. However, one of the more recent cultures from a positive hepatoma showed a few clumps of fluorescent cells in contrast to the single cells seen in all the others.

Morphologically all the cells were fibroblast-like (Fig. 4). Cultures from the malignant tumours showed greater variation in cell size with slightly more mitotic figures. The large sometimes multinucleated brownish granular cells seen with the phase contrast microscope in the first few weeks of culture could not be identified by immunofluorescence. They were seen after staining with Giemsa in some of the slides.

The supernatants from the culture chambers of these cells, collected over various intervals during feeding and before this experiment, were tested without concentration by the double diffusion technique. No precipitin lines were seen in any. Further studies on the concentrated supernatants are presently in progress.

*Homogenate of neonatal liver*

The homogenate and supernatant I gave precipitin lines with rabbit antiserum to human AFP. Phase contrast microscopy of supernatant I showed many intact cells, free nuclei and organelles that appeared as granules. On centrifuging at 800 *g* and 4° C. for 30 minutes, the cells were sedimented and the supernatant clear. On sonication and centrifugation at 800 *g* and 4° C. for 30 minutes, three zones were obtained: namely—a clear supernatant with organelles and cell membrane fragments, a middle white nuclear zone and a deposit of a few intact cells and debris. Ten-fold concentration of the three zones and testing of each gave a precipitin line only with the supernatant. A similar experiment on a control liver from an adult patient who died in a road accident failed to give any precipitin line.

---

EXPLANATION OF PLATES

FIG. 1.—Post mortem liver section from a 5-hour-old male neonate that was delivered after 40 weeks gestation. There is diffuse homogenous cytoplasmic fluorescence predominantly of the periportal hepatocytes, for AFP. × 100. Indirect immunofluorescent technique.

FIG. 2.—Post-mortem liver section from a 45-year-old man with AFP seronegative hepatocellular carcinoma in a non-cirrhotic liver. Cytoplasmic fluorescence is absent except in focal areas due to autofluorescent granules. Occasional parasinusoidal fluorescence is present. Indirect immunofluorescent technique. × 100.

FIG. 3.—19 days tissue culture cells from needle liver biopsy of 24-year-old man with AFP seropositive hepatocellular carcinoma. One fibroblast-like cell shows diffuse cytoplasmic fluorescence. Indirect immunofluorescent technique. × 450.

FIG. 4.—26 days tissue culture cells from needle liver biopsy of a 36-year-old woman with AFP seropositive hepatocellular carcinoma. Morphologically the cells are spindle-shaped (fibroblast-like). May-Grünwald-Giemsa stain. × 180.

Smith, Francis, Edington and Williams

2

3

4

Smith, Francis, Edington and Williams

### DISCUSSION

It has been demonstrated that liver cells synthesize AFP. Irlin *et al.* (1966) used tissue culture cells of transplantable ascitic hepatoma and double diffusion. Abelev and Bakirov (1967) demonstrated AFP with the aid of immunoautoradiography in the livers of newly born mice and rats and Luria *et al.* (1969) with organ culture of embryonal mouse liver. Van Furth and Adinolfi (1969) used human fetal livers *in vitro* by double diffusion and immunoautoradiography. The indirect immunofluorescent technique confirmed these findings in this study. The distribution of the cells that contain AFP and its intracellular localisation has not been commented on, as far as we know. The presence of AFP in the fetal, neonatal and some hepatoma livers only in focal hepatocytes was an unexpected observation. It had been presumed that all the liver cells would secrete AFP. That only about 20% of the hepatocytes secrete AFP in the 28 weeks fetus seems to justify the suggestion of Tatarinov (1965*b*) and Sankale *et al.* (1970) that there are two specific types of liver cells. There are those that are intrinsically capable of producing AFP and others that are not, in the same liver. If in neoplasia AFP positive liver cell carcinomas originate from cells imbued with the capacity to produce AFP, it would be expected that all the malignant hepatocytes in an AFP positive patient would have AFP. This was contrary to our observation. It thus appears that putative AFP positive and negative cells become malignant concurrently or that the intrinsic AFP positive cells can give rise to both AFP positive and negative hepatocytes during neoplasia. If in the fetus and neonate there are thus primary AFP positive and primary AFP negative hepatocytes, it is postulated that the AFP positive fetal hepatocytes either die out or are transformed to secondary AFP negative hepatocytes in the adult. During neoplasia in the fetus or neonate the primary AFP positive hepatocyte may give rise to AFP positive or negative hepatoma. In the adult the primary AFP negative cells give rise to only AFP negative liver cell carcinoma whilst the secondary AFP negative cells may be retransformed to AFP positive hepatocellular carcinoma or may remain as AFP negative ones. If with maturation all AFP positive cells die out, some AFP negative hepatocytes must transform to produce AFP in neoplasia. However if all hepatocytes are originally primarily AFP positive, then all AFP negative cells are only secondarily so.

During malignant transformation there is loss of normal lobular architecture. It is not surprising therefore that the periportal distribution of AFP containing hepatocytes seen in the fetus and neonate is absent in the carcinomatous liver. However, it is possible that in AFP secreting liver cell carcinomas the first neoplastic group of cells are those derived from the periportal areas. Whether all the hepatocytes of the fetus and hepatomas produce AFP but do so at different rates or in different zones of the lobules at different times is not elucidated by this study.

The particular subcellular organelle where AFP is produced might be elucidated by differential ultracentrifugation. Some proteins have been shown to be synthesised in the rough endoplasmic reticulum of the cytoplasm (Loewy and Siekevitz, 1966). Others are associated with mitochondria (Perlman and Penman, 1970). Generally, globulins are synthesised by cells of the reticuloendothelial system (Popper and Schaffner, 1957; Hadziyannis *et al.*, 1969). It is therefore also interesting to find AFP, whose molecular weight is close to that of albumin, being synthesised in liver cells *per se* and not in the reticular cells of the liver.

Because of the transient nature of AFP, its site of production at the subcellular level may differ from that of the more persistent proteins. Our experiments only confirm the intracellular presence of AFP.

Gitlin and Boesman (1967) and Van Furth and Adinolfi (1969) observed that AFP is at its maximum concentration in the fetus of 20 weeks gestation. Several possibilities could account for this. There may be more cells producing AFP at this time with gradual depletion of these specific cells as the fetus grows. It may be that the metabolic activity of the fetus is maximum as this period. The larger size of the fetus as it grows older may cause a relative dilution of AFP.

Irlin *et al.* (1966) observed that during the early stages of cultivation of mouse transplantable ascitic hepatoma, AFP, albumin and B 2-1 globulin were demonstrated in the tissue culture medium for up to 13 weeks. In the course of subsequent cultivation, the production of AFP ceased in 16 weeks while albumin and B 2-1 globulin were present in the medium during the whole period of cultivation which was up to 69 weeks. In our experiments human AFP was detectable in the cultured cells by immunofluorescence 50 days after primary cultivation. The rate of catabolism of AFP is unknown. It is possible that some of the AFP detected may be the remnant in the originally explanted cells rather than AFP that is freshly synthesized. Work is in progress to find out how long AFP secretion can persist *in vitro.* Irlin *et al.* (1966) also found that transplantation of some mouse ascitic hepatoma that had stopped secreting AFP *in vitro* into a new set of mice reactivated its synthesis *in vivo.*

Although Gitlin and Boesman (1967) and Van Furth and Adinolfi (1969) showed that AFP is not produced by other fetal tissues apart from the liver, a confirmation of their findings by the immunofluorescent technique is warranted. This was not done during this study.

The indirect immunofluorescent technique is as sensitive as the double diffusion method for the detection of AFP. Whether it is more sensitive is difficult to say from this study because of the small numbers involved. However, from the small percentage of AFP secreting cells, a false negative result could be obtained by sampling error especially from needle biopsy specimens.

It is well known that serum proteins can leak passively into cells after cell death (Kent, 1969). Most of this work was done on autopsy material and this could be said to account for the fluorescence seen. The striking distribution of specific fluorescence in the fetal and neonatal liver, the presence of fluorescent hepatocytes in two liver biopsies from live AFP positive hepatoma patients, and the presence of AFP in tissue culture cells from similar patients strongly suggest that the AFP detected was synthesised by the cells.

This work is supported by the Rockefeller foundation through the University of Ibadan Medical Research Training Fellowship. It is part of a thesis in preparation by J.A.S. We are indebted to Dr. B. O. Osunkoya and Mr. T. Ilori for help with the tissue culture. Professor G. Abelev, Dr. Ph. Sizaret and Dr. J. Uriel kindly supplied the reference rabbit antisera and Professor V. Houba provided the conjugated antiserum. Mr. E. A. Ogiugo cut the cryostat sections. The WHO Immunology Centre made all its facilities available to us. The Medical Illustration Unit helped with the photomicrographs.

### REFERENCES

ABELEV, G., ASSECRITOVA, I., KRAEVSKY, N., PEROVA, S. AND PERVODCHIKOVA, N.—(1967) *Int. J. Cancer*, **2**, 551.

ABELEV, G. I. AND BAKIROV, R. D.—(1967) *Vop. med. Khim.*, **13**, 378.

FOLI, A. K., SHERLOCK, S. AND ADINOLFI, M.—(1969) *Lancet*, ii, 1267.

GITLIN, D. AND BOESMAN, M.—(1967) *Comp. Biochem. Physiol.*, **21**, 327.

HADZIYANNIS, S., FEIZI, T., SCHEUER, P. J. AND SHERLOCK, S.—(1969) *Clin. exp. Immunol.*, **5**, 499.

HOLBOROW, E. J. AND JOHNSON, G. D.—(1967) in ' Handbook of Experimental Immunology '. Edited by D. M. Weir. Oxford and Edinburgh (Blackwell) p. 582.

IRLIN, I. S., PEROVA, S. D. AND ABELEV, G. I.—(1966) *Int. J. Cancer*, **1**, 337.

KENT, S. P.—(1969) *Archs Path.*, **88**, 407.

LOEWY, A. G. AND SIEKEVITZ, P.—(1966) ' Cell Structure and Function '. New York (Holt, Rinehart and Winston) p. 159.

LURIA, E. A., BAKIROV, R. D., YELISEYEVA, T. A., ABELEV, G. I. AND FRIEDENSTEIN, Y. —1969) *Expl Cell Res.*, **54**, 111.

MASOPUST, J., KITHIER, K., RÁDL, J., KOUTECKY, J. AND KOTAL, L.—(1968) *Int. J. Cancer*, **3**, 364.

PERLMAN, S. AND PENMAN, S.—(1970) *Nature, Lond.*, **227**, 133.

POPPER, H. AND SCHAFFNER, F.—(1967) ' Liver: Structure and Function '. New York (McGraw Hill) p. 99.

SANKALE, M., SOW, A. M. AND BAO, O.—(1970) *Ghana med. J.*, **9**, 44.

SULLMAN, S. F., ZUCKERMAN, A. J., TAYLOR, P. F. AND STUBB, J.—(1968) *J. clin. Path.*, **21**, 794.

TATARINOV, YU. S.—(1965*a*) *Vop. med. Khim.*, **11**, 17 (translated in *Fedn Proc. Fedn Am. Socs exp. Biol.*, 1966, **25**, T344).—(1965*b*) Cited by G. I. Abelev, 1968, *Cancer Res.*, **28**, 1344.

TAYLOR, P. F., ZUCKERMAN, A. J. AND FARROW, C. F.—(1969) *J. clin. Path.*, **22**, 701.

VAN FURTH, R. AND ADINOLFI, M.—(1969) *Nature, Lond.*, **222**, 1296.

WILLIAMS, A. I. O. AND MCFARLANE, H.—(1968) *Clin. exp. Immun.*, **3**, 953.

ZUCKERMAN, A. J.—(1967) *J. clin. Path.*, **20**, 675.

ZUCKERMAN, A. J., TSIQUAYE, K. N. AND FULTON, F.—(1967) *Br. J. exp. Path.*, **48**, 20.


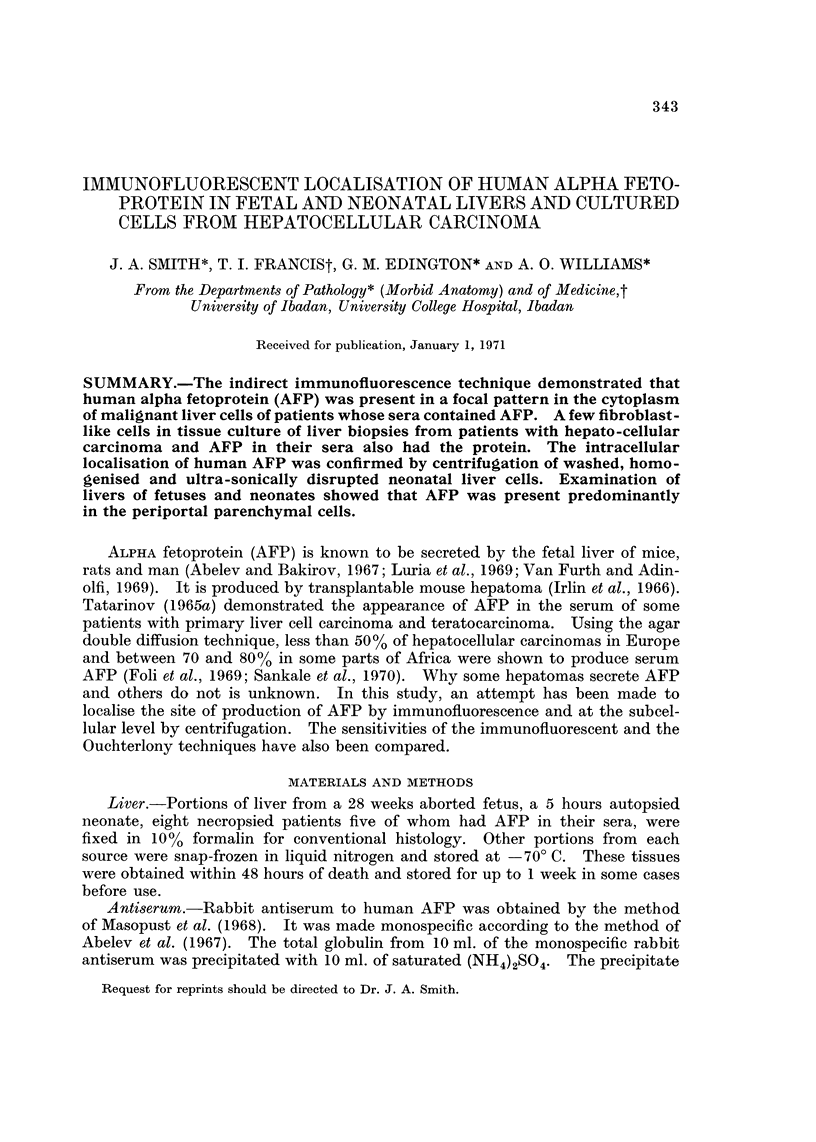

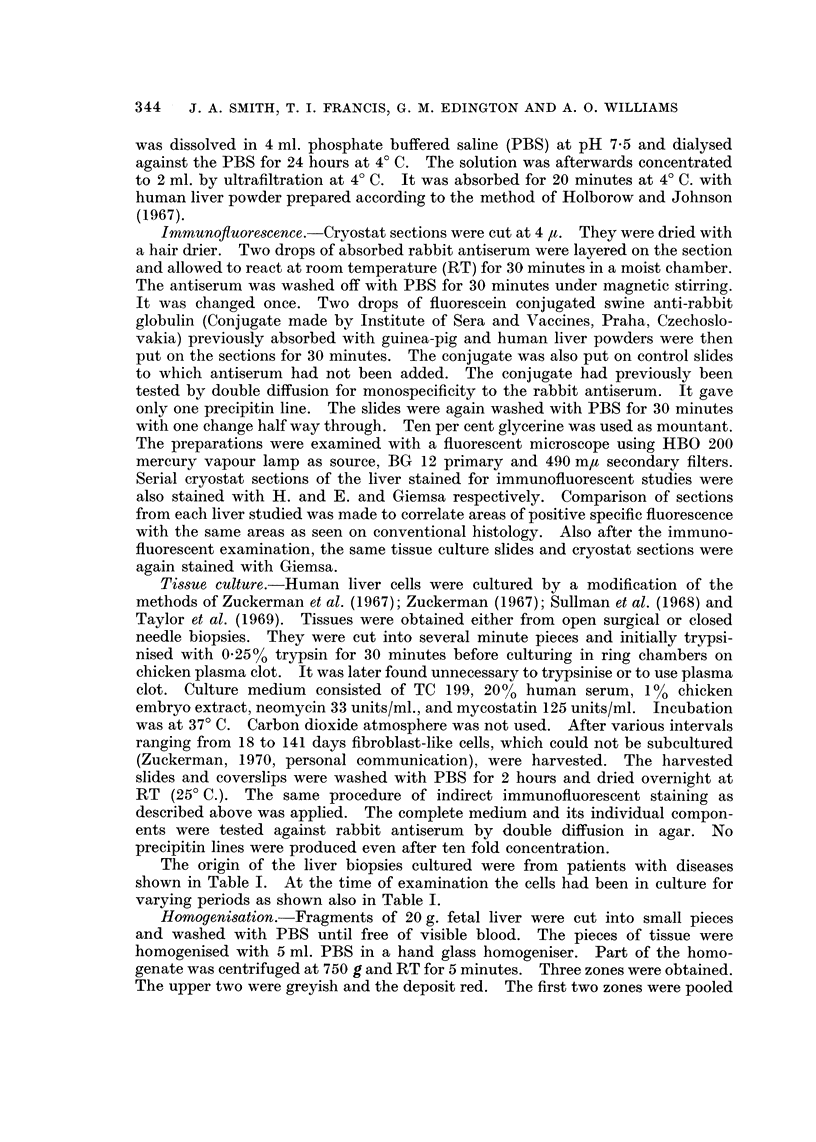

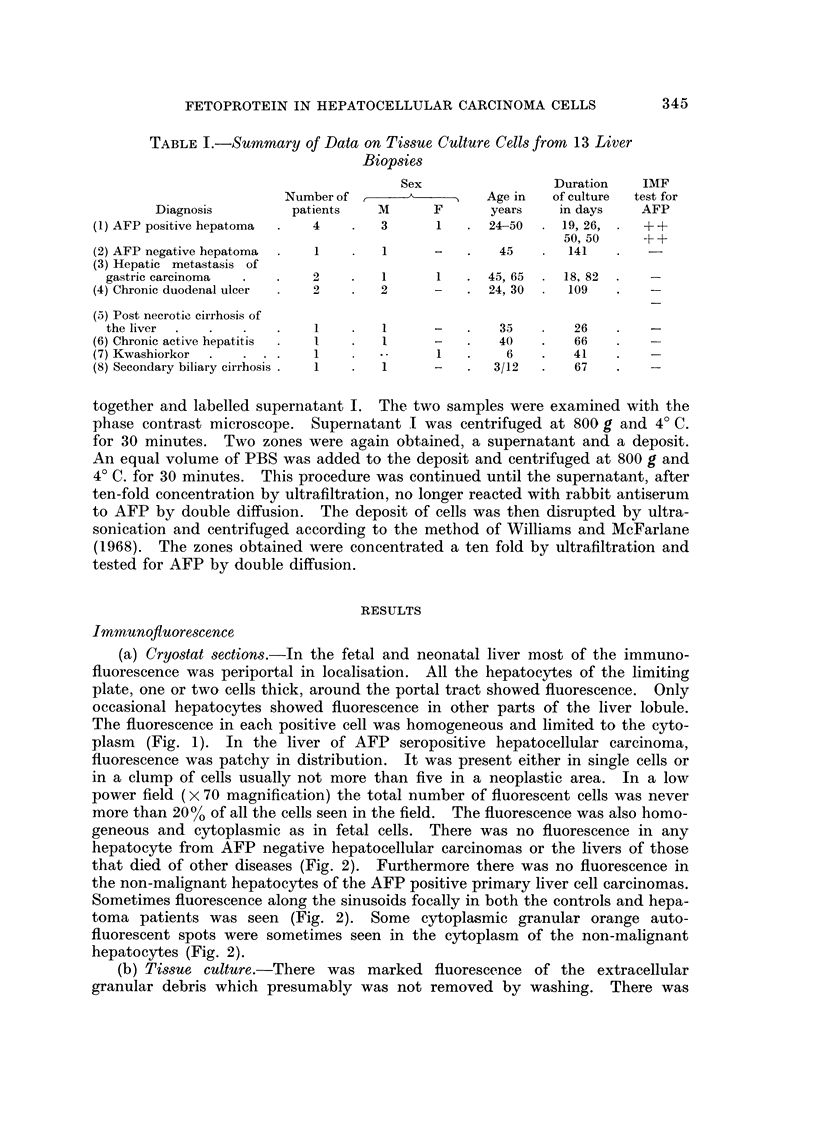

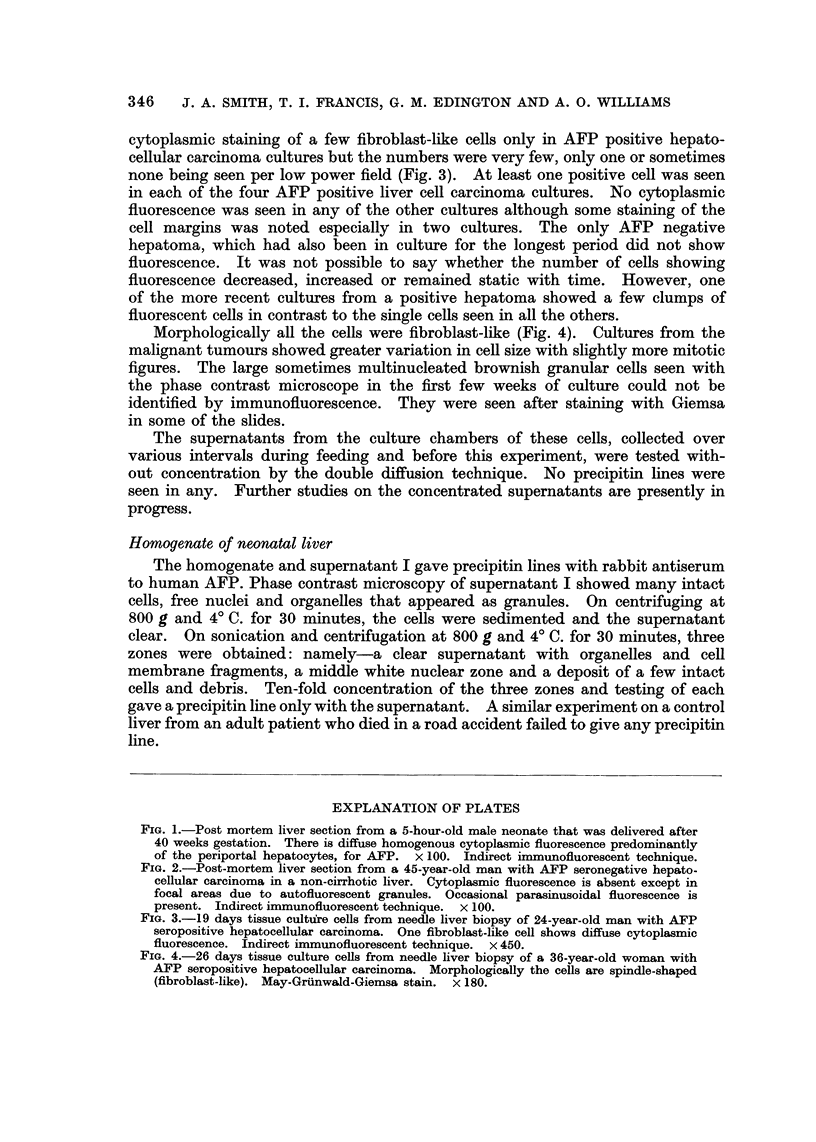

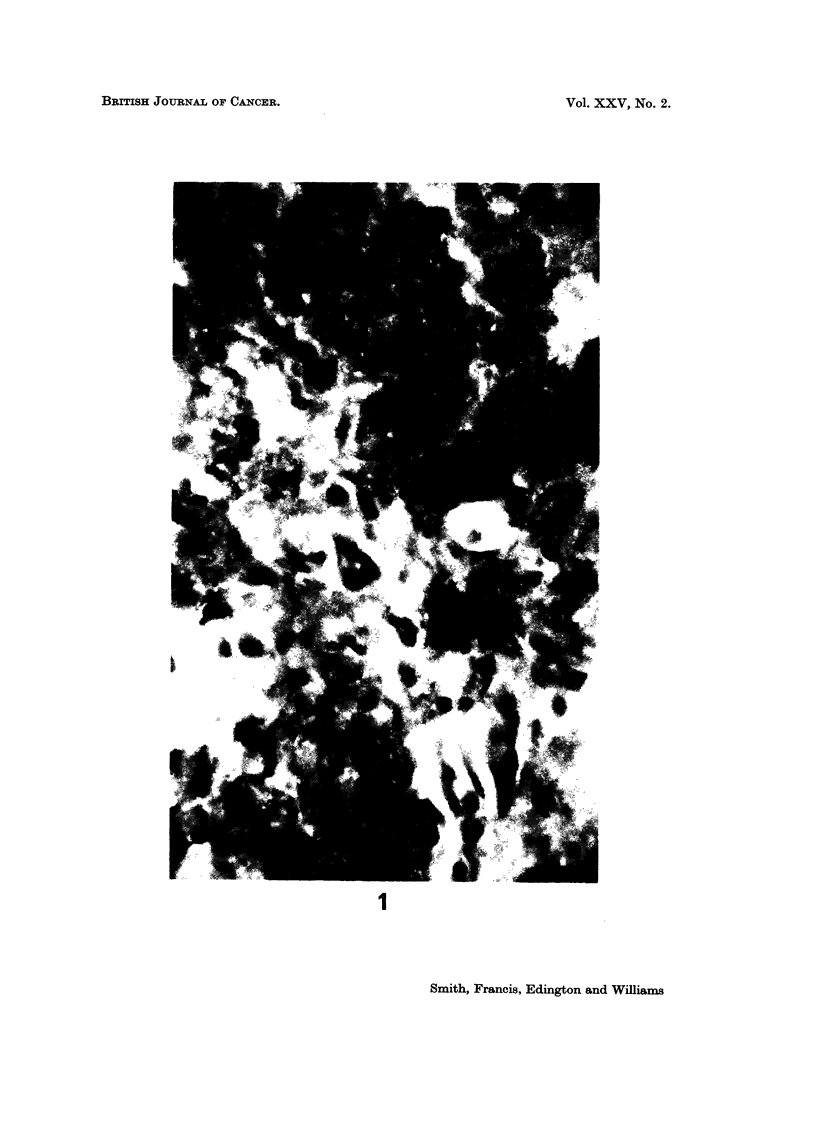

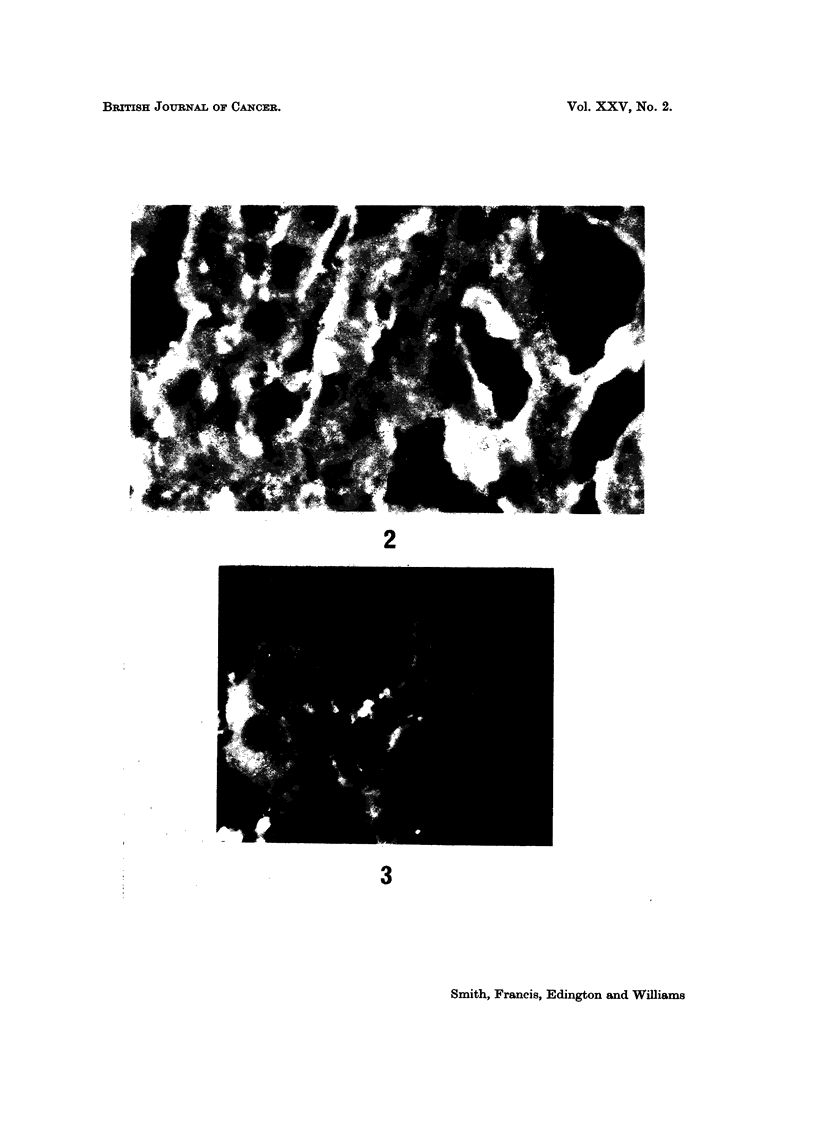

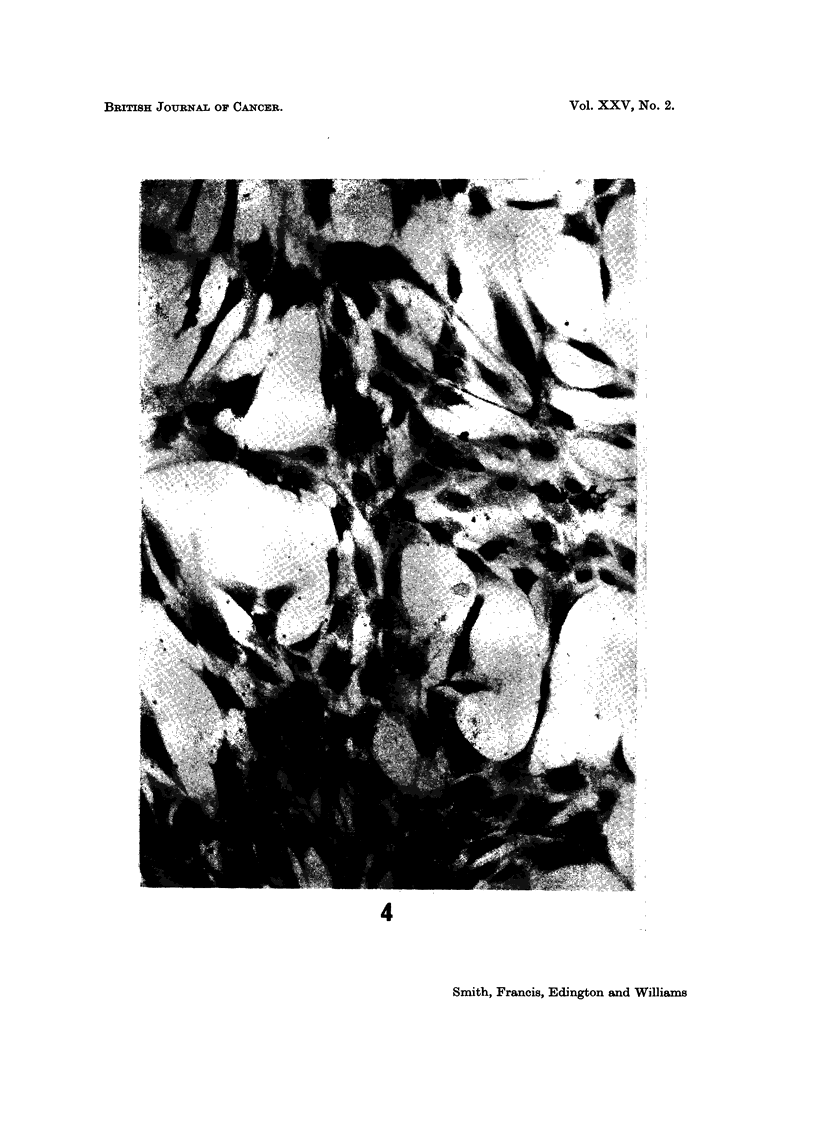

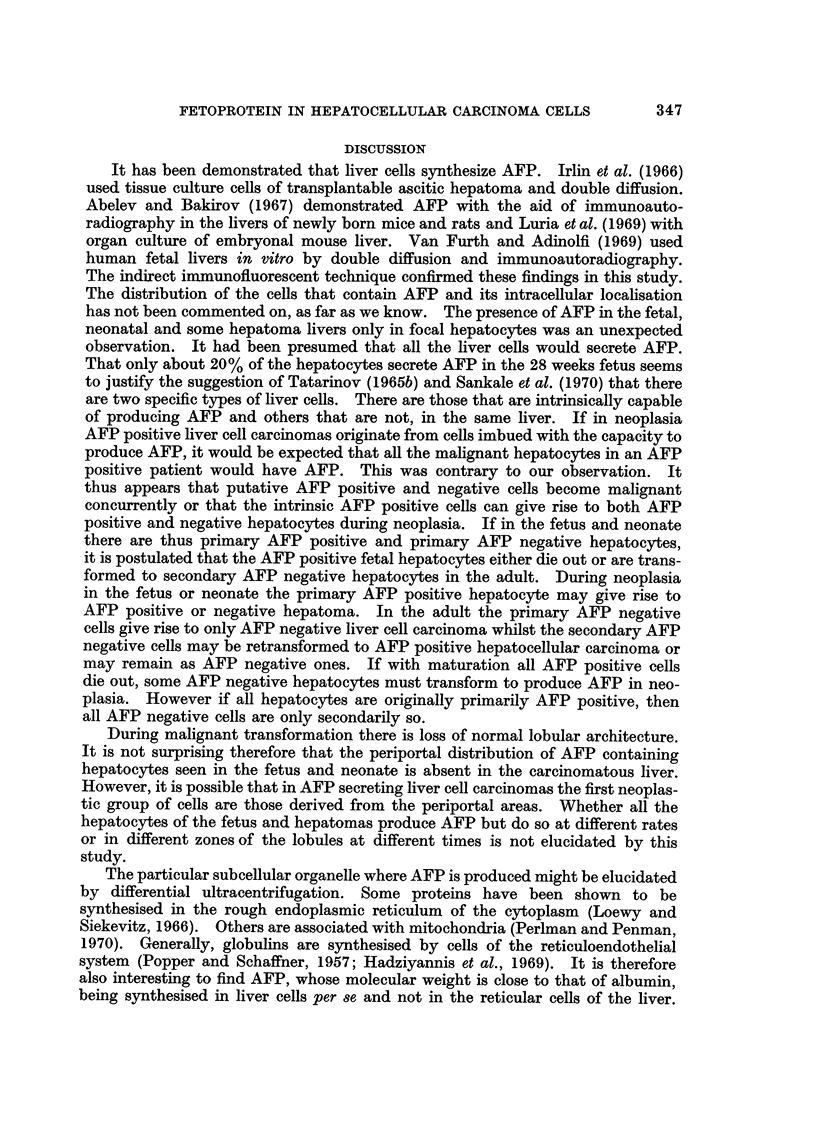

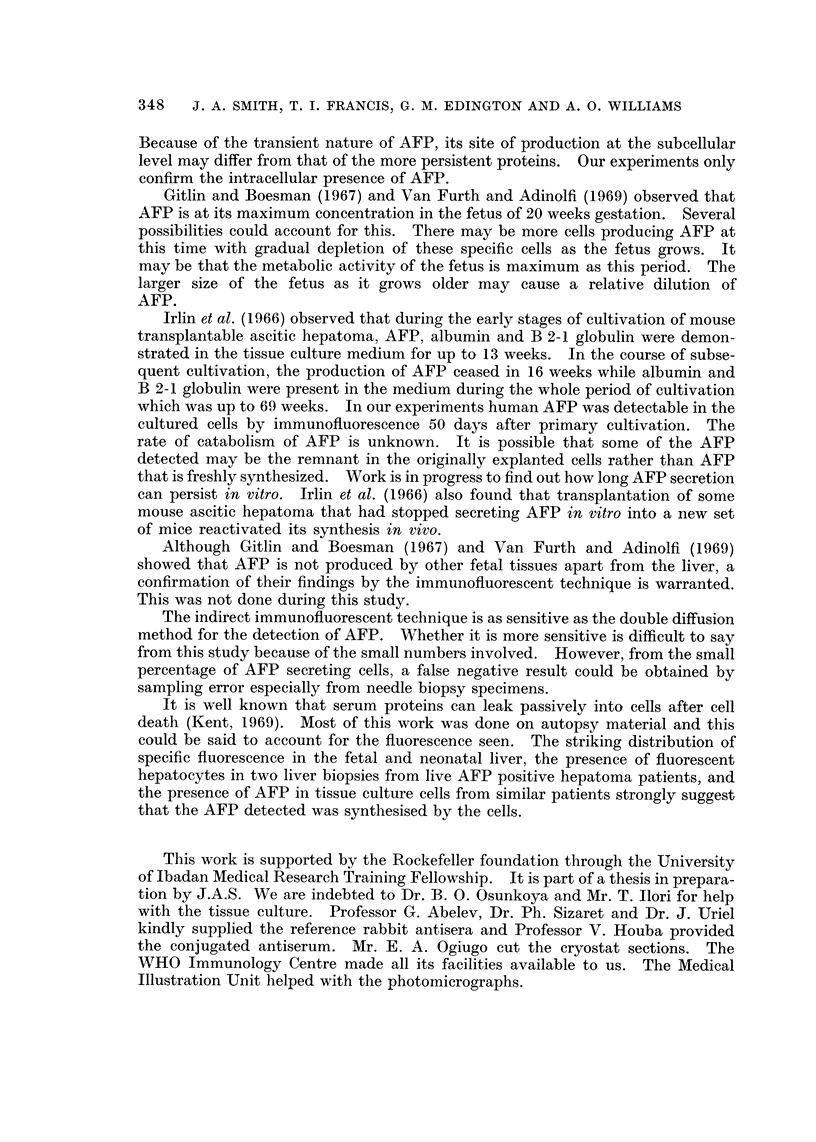

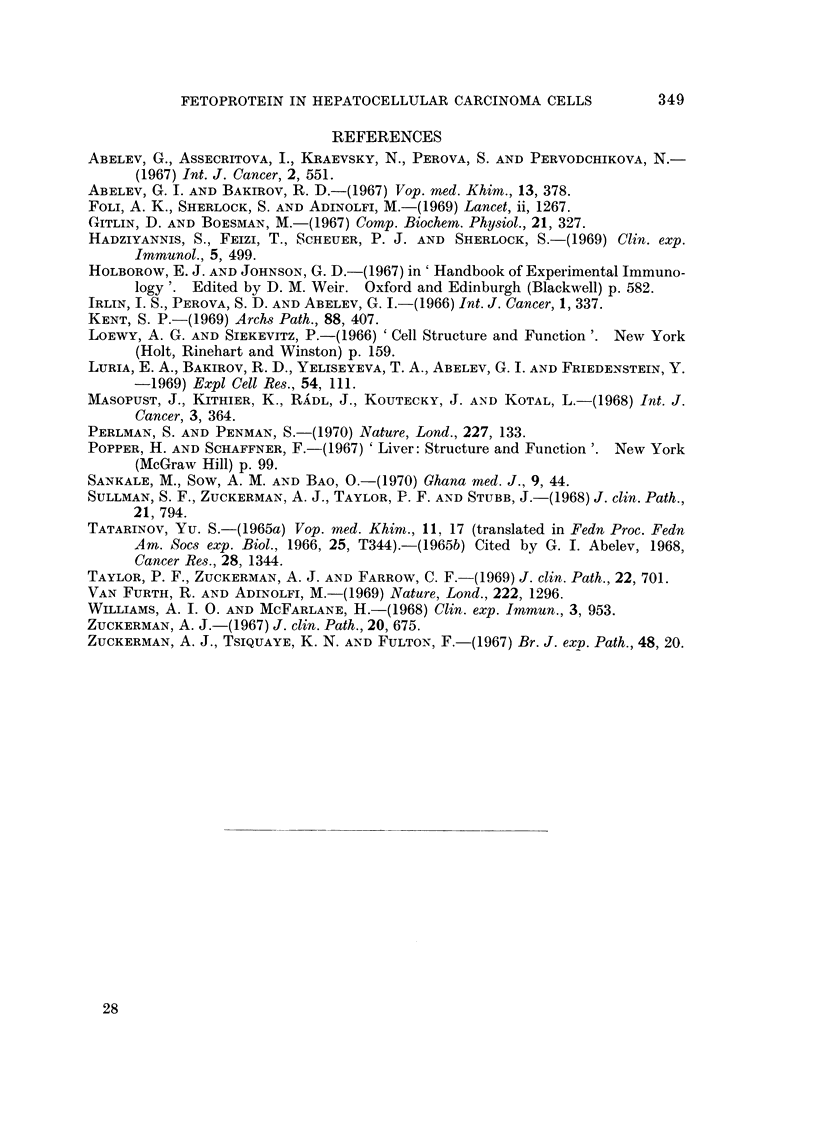


## References

[OCR_00385] Abelev G. I., Assecritova I. V., Kraevsky N. A., Perova S. D., Perevodchikova N. I. (1967). Embryonal serum alpha-globulin in cancer patients: diagnostic value.. Int J Cancer.

[OCR_00388] Abelev G. I., Bakirov R. D. (1967). Sintez émbrional'nykh antigenov syvorotki pechen'iu in vitro.. Vopr Med Khim.

[OCR_00428] Abelev G. I. (1968). Production of embryonal serum alpha-globulin by hepatomas: review of experimental and clinical data.. Cancer Res.

[OCR_00390] Gitlin D., Boesman M. (1967). Fetus-specific serum proteins in several mammals and their relation to human alpha-fetoprotein.. Comp Biochem Physiol.

[OCR_00392] Hadziyannis S., Feizi T., Scheuer P. J., Sherlock S. (1969). Immunoglobulin-containing cells in the liver.. Clin Exp Immunol.

[OCR_00406] Luria E. A., Bakirov R. D., Yeliseyeva T. A., Abelev G. I., Friedenstein A. Y. (1969). Differentiation of hepatic and hematopoietic cells and synthesis of blood serum proteins in organ cultures of the liver.. Exp Cell Res.

[OCR_00410] Masopust J., Kithier K., Rádl J., Koutecký J., Kotál L. (1968). Occurrence of fetoprotein in patients with neoplasms and non-neoplastic diseases.. Int J Cancer.

[OCR_00416] Perlman S., Penman S. (1970). Protein-synthesizing structures associated with mitochondria.. Nature.

[OCR_00424] Sullman S. F., Zuckerman A. J., Taylor P. F., Stubbs J. (1968). Tissue culture of adult liver biopsies.. J Clin Pathol.

[OCR_00431] Taylor P. E., Zuckerman A. J., Farrow L. J. (1969). Culture of needle biopsies of the liver from patients with suspected hepatitis.. J Clin Pathol.

[OCR_00432] Van Furth R., Adinolfi M. (1969). In vitro synthesis of the foetal alpha 1-globulin in man.. Nature.

[OCR_00434] Williams A. I., McFarlane H. (1968). Malarial antigen from human brain.. Clin Exp Immunol.

